# Landmark clinical observations and immunopathogenesis pathways linked to HIV and *Cryptococcus* fatal central nervous system co‐infection

**DOI:** 10.1111/myc.13122

**Published:** 2020-06-19

**Authors:** Samuel Okurut, David R. Boulware, Joseph Olobo, David B. Meya

**Affiliations:** ^1^ Research Department Infectious Diseases Institute Makerere University Kampala Uganda; ^2^ Department of Microbiology School of Biomedical Sciences College of Health Sciences Makerere University Kampala Uganda; ^3^ Division of Infectious Diseases and International Medicine Department of Medicine University of Minnesota Minneapolis Minnesota; ^4^ Department of Immunology and Molecular Biology School of Biomedical Sciences College of Health Sciences Makerere University Kampala Uganda; ^5^ Department of Medicine School of Medicine College of Health Sciences Makerere University Kampala Uganda

**Keywords:** B‐cell immune regulation, brain fibrosis, central nervous system evasion, HIV‐associated cryptococcal meningitis co‐infection, Human *Cryptococcus* infection, immune activation, pathogenesis, PD‐1/PD‐L1 immune regulation, treatment outcome

## Abstract

Cryptococcal meningitis remains one of the leading causes of death among HIV‐infected adults in the fourth decade of HIV era in sub‐Saharan Africa, contributing to 10%–20% of global HIV‐related deaths. Despite widespread use and early induction of ART among HIV‐infected adults, incidence of cryptococcosis remains significant in those with advanced HIV disease. *Cryptococcus* species that causes fatal infection follows systemic spread from initial environmental acquired infection in lungs to antigenaemia and fungaemia in circulation prior to establishment of often fatal disease, cryptococcal meningitis in the CNS. *Cryptococcus* person‐to‐person transmission is uncommon, and deaths related to blood infection without CNS involvement are rare. Keen to the persistent high mortality associated with HIV‐cryptococcal meningitis, seizures are common among a third of the patients, altered mental status is frequent, anaemia is prevalent with ensuing brain hypoxia and at autopsy, brain fibrosis and infarction are evident. In addition, fungal burden is 3‐to‐4‐fold higher in those with seizures. And high immune activation together with exacerbated inflammation and elevated PD‐1/PD‐L immune checkpoint expression is immunomodulated phenotypes elevated in CSF relative to blood. Lastly, though multiple *Cryptococcus* species cause disease in this setting, observations are mostly generalised to cryptococcal infection/meningitis or regional dominant species (*C neoformans* or *gattii complex*) that may limit our understanding of interspecies differences in infection, progression, treatment or recovery outcome. Together, these factors and underlying mechanisms are hypotheses generating for research to find targets to prevent infection or adequate therapy to prevent persistent high mortality with current optimal therapy.

## 1 INTRODUCTION

The central nervous system (CNS) (brain, cerebrospinal fluid (CSF) and spinal cord) provides a formidable niche for disseminated fatal cryptococcal meningitis.^1^, ^2^ Cryptococcal meningitis is an acute fungal disease caused by an encapsulated yeast of the genus *Cryptococcus*.^3^ *Cryptococcus* emerged from the environment to cause disease in man and the species that cause fatal infection have high preference to infect the CNS to cause meningoencephalitis in individuals with ensuing immunosuppression.^2^, ^4^, ^5^, ^6^ Several species of *Cryptococcus* exist with *Cryptococcus neoformans* complex and *Cryptococcus gattii* complex leading infection in those with an underlying immunosuppression.^3^, ^5^, ^7^, ^8^, ^9^, ^10^, ^11^, ^12^, ^13^ The *C neoformans* species complex comprise of *C neoformans* sensu* stricto* that causes 60%‐90% of HIV‐associated cryptococcal meningitis together with *C deneoformans* and hybrids between both species.^8^, ^9^, ^10^, ^11^, ^14^, ^15^ Prior to individual species characterisation, *C gattii* complex was known to cause disease mostly among individuals without HIV infection.^6^, ^8^ But to date increasing reports of different species of *C gattii* complex are being documented to cause disease among HIV immunocompromised patients globally.^8^ The *C. gattii* species complex includes *C gattii* sensu* stricto* (AFLP4/VGI), *C deuterogatii* (AFLP6/VGII), *C bacillisporus* (AFLP5/VGIII), *C tetragattii* (AFLP7/VGIV) and *C decagattii* (AFLP10/VGIII and VGIV).^7^, ^10^, ^16^, ^17^, ^18^ Thus, the limitations of existing diagnostic tools in common clinical use that are unable to identify species‐related infection may limit our understanding of information related to species‐specific infection, pathogenesis and disease outcome. The species‐related information may be relevant in designing treatment strategies amidst high residual cryptococcal meningitis‐related deaths with optimal use of antifungal drugs to treat those co‐infected with HIV.

Among HIV‐infected adults (>18 years of age), cryptococcal meningitis is diagnosed in CSF 7‐28 days from onset of symptoms.^19^, ^20^, ^21^, ^22^, ^23^, ^24^ However, early symptoms including fever and headache may complicate early diagnosis and delay antifungal treatment in regions with other endemic pathogens that present with similar symptoms.^20^, ^21^, ^25^, ^26^ Cryptococcosis results in 20%‐40% HIV‐related deaths worldwide.^23^, ^27^ Among HIV‐infected adults, CD4 T‐cell count < 100 cell/µL is one of the risk factors for cryptococcosis.^20^, ^23^, ^28^ However, despite attempts to restore and maintain immune response with early antiretroviral therapy (ART) among HIV‐infected individuals with higher CD4 T cells, some persons still present with high incidence of HIV‐associated cryptococcosis.^23^, ^29^, ^30^, ^31^, ^32^ Surprisingly, among regions with a high incidence of HIV‐associated cryptococcosis, one would relate incidence of infection with frequent yeast exposure from the environment. But rare infection among healthy individuals in this regions alters this speculation.^33^

Hence, the challenges in the pathogenesis of human *Cryptococcus* infection have led to the incidence of cryptococcosis remaining significant over the last three to four decades of the HIV/AIDS epidemic especially in sub‐Saharan Africa.^21^, ^23^, ^30^, ^34^, ^35^ Indeed, persistent high mortality from HIV‐associated cryptococcosis occurs with the use of optimal antifungal drugs and HAART for the treatment of those co‐infected.^20^, ^21^, ^22^, ^23^ It remains unclear what constitutes a translational mechanism to attenuate this high mortality among those with HIV‐associated cryptococcosis. Of note, in large cryptococcosis cohorts, factors associated with treatment failure have been inconsistently reported.^20^, ^21^, ^22^, ^23^, ^24^, ^36^, ^37^ Hence, this review aims to point the field to the cryptococcosis immunopathogenesis factors and pathways to be targeted in further investigations to prevent infection or to alter persistent poor treatment outcome.

## 2 PATHOGENESIS OF HUMAN CRYPTOCOCCOSIS INFECTION

The initial *Cryptococcus* infection is postulated to occur in the lungs where alveolar macrophages, (the primary cells to encounter *Cryptococcus*) together with activated Th1 and Th17 cells form the cornerstone of protection.^2^, ^27^ In theory, recruited activated immunocytes surround infected primary macrophages to form a granuloma.^27^, ^38^ During this process, the primary infection is contained through phagocytosis leading to complete resolution of infection or through evasion of phagocytosis leading to establishment of latency. The evidence for cryptococcosis resolution without disease onset (aborted infection) and/or latency is based on the presence of *Cryptococcus*‐positive binding antibodies in children that demonstrate early exposure to the fatal yeast.^39^ Among children and adults who develop active cryptococcosis, progression follows an onset of HIV and other immunosuppressive disease condition including HIV advanced disease.^6^, ^14^, ^40^ Hence, latent *Cryptococcus* infection may persist for a lifetime among *Cryptococcus* exposed individuals without an underlying immunosuppression trigger.^23^, ^41^, ^42^

It is proven by molecular diagnostics (eg MLST on cultures) that *Cryptococcus tetragattii* (known to only occur in Africa/India) can be dormant or latent for 20‐30 years after exposure, for example in immigrants who develop cryptococcal meningitis with strains from country of origin after acquiring HIV in the country of residence.^6^, ^40^, ^43^ Similarly, *C deuterogattii* infection (Vancouver Island outbreak‐causing *Cryptococcus* species) is observed to cause disease among tourists who developed disease years after a touristic visit to the affected area in North America with several European case reports of people who had underlying diseases, like in systemic lupus erythematosus.^6^, ^8^, ^43^, ^44^ Thus, without an onset of HIV immune suppression mostly in sub‐Saharan Africa and Asia Pacific regions and without onset of malignancies and cancer treatment, solid organ transplant conditioning in the developed world, and prolonged hospitalisation, latent *Cryptococcus* infection may potentially not progress to fatal cryptococcal meningitis.^45^, ^46^, ^47^, ^48^ Other factors associated with onset of active *Cryptococcus* infection include the following: loss of quality multifunctional CD4 T cells among HIV‐infected individuals,^49^, ^50^ loss of IgM + memory B cells (innate‐like IgM producing memory B cells) among HIV‐infected adults,^51^ use of B cell‐depleting antibody therapies (rituximab and infliximab) among cancer patients,^47^, ^52^, ^53^ defects in the FC‐γ receptor polymorphism^54^, ^55^, ^56^ and fungal evasion of phagocytosis (immune escape).^38^, ^57^, ^58^

Conversely, whether fatal systemic cryptococcosis is due to new infection or reactivated latent infection is not clearly understood.^34^, ^59^ However, *Cryptococcus* genotyping studies from clinical isolates of emigrants with *Cryptococcus* strains from endemic regions support both the latent and new environmental acquired *Cryptococcus* infection causality theories.^5^, ^60^ That, individuals who travel and developed cryptococcosis infection with strains endemic to their region support the latent infection theory. Clearly, from European multilocus sequence typing (MLST)—studies, as above mentioned with immigrants from, for example Africa who developed *C tetragattii* infection ~26 years after immigrated from Zambia to Sweden where the patient acquired HIV followed by cryptococcal meningitis, the isolated strains were identical to the *C tetragattii* lineage from Southern Africa.^40^ And that, similar MLST studies in Africa, Americas and Asia among adult travellers and immigrants who develop cryptococcosis infection with strains endemic to regions they travelled or settled support the new infection theory.^6^, ^8^, ^9^ Moreover, person‐to‐person transmission of *Cryptococcus* to cause infection is rare.^6^, ^8^, ^9^, ^11^, ^43^ Thus, the limited cryptococcosis person‐to‐person transmission limits genetic recombination between *Cryptococcus* species and maintains environmental acquired *Cryptococcus* strain clonality that is easily traced to the origin of acquisition. And the lack of person‐to‐person transmission of cryptococcus limits public attention towards this debilitating disease among HIV immune compromised patients. Thus, *Cryptococcus* host exposure dynamics from environment to cause infection together with host immunogenetic factors and mechanisms may influence onset of fatal cryptococcosis.^2^, ^10^, ^12^, ^13^, ^16^, ^17^, ^18^, ^61^ And deeper investigation may leverage the mechanisms of infection to alter onset of fatal cryptococcal meningitis or prevent deaths among those undergoing treatment.

## 3 *CRYPTOCOCCUS* CENTRAL NERVOUS SYSTEM INFECTION

Recent findings indicate several *Cryptococcus* transmission mechanisms are used by the yeast to infect the CNS. The trojan horse model, where *Cryptococcus*‐infected macrophages, traverse the blood‐brain barrier (BBB) to infect the central nervous system.^62^ This trojan horse mechanism is also observed to facilitate HIV transmission across the BBB in response to intrathecal CCL2 chemokine stimulation.^63^ And, restriction of adhesion molecules (JAM‐A and ALCAM and chemokine receptors (CCR2 and CCR5) with inhibitory antibodies restricted monocytes trafficking to the CSF lowered CSF HIV viraemia.^63^ Transcytosis is another mechanism, where *Cryptococcus,* with the help of cellular binding motifs and adhesion molecules, manoeuvres through interstitial cellular spaces of the BBB and the brain parenchyma to infect the CNS. Dendritic cells are also associated with the transcytosis mechanism of compartmentalised pathogen transmission. Lastly, paracytosis is another mechanism where *Cryptococcus* with the help of secreted proteases digest its way through the cells lining the BBB to gain access of the CNS.^62^, ^64^ Thus, interruption of the pathways that aid *Cryptococcus* access to the CNS where it establishes fatal disease could alter clinical outcomes.^23^, ^45^, ^65^

**Figure 1:**
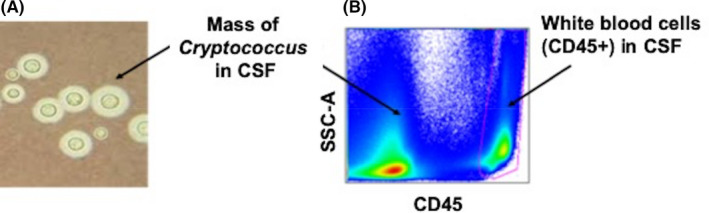
Flow cytometry representation of the cellular lineage of fresh cerebrospinal fluid from a subject with HIV‐associated cryptococcal meningitis. A, magnified encapsulated cryptococcal cells, India ink staining and viewed under a light microscope Curtsey CDC/Dr Leanor Haley (https://en.wikipedia.org/wiki/Cryptococcus_neoformans#/media/File:Cryptococcus_n eoformans_using_a_light_India_ink_staining_preparation_PHIL_3771_lores.jpg). B, CSF cell pellet stained with CD45 fluorescent labelled antibodies and analysed using a flow cytometer. Here, CD45 is a pan white blood cell marker used to discriminate white blood cells from a mass of *Cryptococcus* cells

The evidence that *Cryptococcus* is predominant in the CSF compartment of individuals with cryptococcal meningitis (Figure 1),^24^, ^66^ suggests that the fungus influences host immunological and treatment outcomes via its effects on the central nervous system (brain, spinal cord, and CSF). Thus, the predominance of the facultative extracellular *Cryptococcus* in the closed CSF interacting with immunocytes may be a translational target to model innate and adaptive immune factors and mechanisms of infection and treatment outcome. But the perturbing question is the elusive role and the lack of clinical relevance of the CSF immunocytes in influencing infection and treatment outcome. Evidence from a Ugandan cohort suggests that some individuals with asymptomatic cryptococcosis present with measurable cryptococcal antigen in blood but not in CSF.^23^, ^29^ And another observation suggests that *Cryptococcus* is unable to proliferate in CSF supernatant from healthy individuals without apparent immunosuppression.^38^ The above two pieces of observations contradict our understanding of the role of CSF in merely supporting establishment of fatal cryptococcosis.

## 4 PD‐1 EXPRESSION ON THE CEREBROSPINAL FLUID IMMUNOCYTES

The immunocytes in the CSF comprise of activated (HLA‐DR/CD38) mature T cells (CD4 and CD8 T cells), monocytes (CD14/CD16; Classical monocytes, Intermediate monocytes and alternative monocytes) and natural killer (NK) cells (CD56/CD16; Bright, dim and negative NK cells).^66^ In addition, the B cells (CD19) in the CSF comprise of activated (CD21 low) and differentiated memory (CD27) and plasmablasts/plasma B cells.^24^ Surprisingly, from the above studies, activation and differentiation of the CSF cellular phenotype seem to be altered by programmed death‐1 (PD‐1; CD279) immune checkpoint receptor and programmed death‐1 ligand (PD‐L1; CD274) expression.^24^, ^66^

The PD‐1 is a ubiquitous immune checkpoint transmembrane molecule that is expressed on lymphoid cells particularly on T follicular helper cells^67^, ^68^ and on cells of the myeloid lineage with B cells, monocyte, macrophages and NK cells being effector cells.^69^, ^70^, ^71^ PD‐1 expression is triggered following induced cellular activation.^72^ The PD‐1 expression is maintained with persistent antigenaemia observed in chronic infections or in slowly resolving infection following treatment success.^73^ PD‐1 interacts with its high‐affinity ligand PD‐L1 (CD274) expressed mostly on antigen presenting cells (monocytes, macrophages and dendritic cells).

PD‐1 expression on CSF B cells, T cells and monocytes is much higher in the CSF compartment compared to the peripheral circulation during cryptococcosis.^24^ Additionally, on monocytes, PD‐L1 expression in human cryptococcosis is equally higher in CSF compared to its expression in peripheral circulation.^66^ Moreover, the majority of CSF immune cells during human cryptococcosis are highly activated compared to those in circulation.^24^, ^66^ Thus, whether the high PD‐1 expression is due to cryptococcal evasion of CNS infection or a host mechanism to downregulate *Cryptococcus* induced CSF/CNS activation remains unclear.

The PD‐1/PD‐L1 interaction results in negative feedback signalling that downregulates activated responses.^74^, ^75^ On effector cells, persistent PD‐1/PD‐L1 interaction results in immune unresponsiveness with consequent immune exhaustion.^76^ Therefore, host PD‐1 induction as a mechanism to circumvent induced pathogen activation may work in synergy against the host to facilitate immune escape by the pathogen to the detriment of the host. For example, shutting down immune activation could facilitate unchecked pathogen replication to the detriment of the host. In addition, exhaustion of immune responses may induce host‐pathogen immune induced tolerance hence accumulation of pathogen host induced damage responses that may lead to host clinical deterioration.^77^ In summary, irrespective of the effector cell, the PD‐1/PD‐L1 interaction inhibits cellular activation, proliferation, antibody production and cytokine expression. Conversely, it is possible that alteration of PD‐1/PD‐L1 interaction by either PD‐1/PD‐L1 blockers restores/reconstitutes the exhausted/inhibited response. Thus, further interrogation of the PD‐1/PD‐L1 pathway in human cryptococcal infection may alter the outcomes of this debilitating disease.^23^, ^34^

## 5 PD‐1 EXPRESSION ON LUNG IMMUNOCYTES

The mucosal lining of the lung provides easy access by the scavenging cells, including the macrophages and dendritic cells to gain access to the inhaled pathogens to influence onset of infection. In mouse models of cryptococcosis, persistent lung infection sustains PD‐1 expression on dendritic cells and on macrophages.^73^ Moreover, sustained PD‐1 expression on the macrophages on cryptococcosis brain mouse models promotes fungal growth through upregulated proliferation of *Cryptococcus*‐infected macrophages, facilitating fungal dissemination.^73^, ^78^ The PD‐1 upregulation further increases activation of microglial cells and promotes Th2 cellular‐activated responses while downregulating Th1‐activated responses.^78^ Of note, application of anti‐PD‐1/PD‐L1 antagonists (PD‐1 blocking antibodies) altered PD‐1‐modulated responses by promoting fungal clearance, upregulating ICOS and XO40 on Th1, Th2, Th17 and regulatory T cells while downregulating IL‐5 and IL‐10 immune regulatory cytokines in the model of cryptococcosis.^73^ In addition, similar responses were observed with IL‐10 blockade in experimental cryptococcal infection.^79^

The IL‐10 modulates immune response in a similar manner as PD‐1 by modulating immune activation, cellular proliferation and cytokine expression in addition to modulation of cellular differentiation.^80^, ^81^, ^82^ Moreover, markers that induce PD‐1 expression on B cells are found to induce IL‐10 expression in B cells through Toll‐like receptor‐9‐mediated mechanism.^72^, ^83^ The PD‐1‐binding antibodies (substitute for PD‐L1) inhibit PD‐1 interaction with its high‐affinity ligand PD‐L1, hence antagonising immune inhibitory activity of PD‐L1 on effector cells and consequently restoring exhausted immune response as observed in the mouse model of lung cryptococcosis infection.^73^ The similar interpretation of PD‐1‐blocking antibodies could be translated to IL‐10 inhibitory (blocking) antibodies in application.

## 6 CENTRAL NERVOUS SYSTEM IMMUNE ACTIVATION AND INFLAMMATION

Immune activation and inflammation are a cornerstone of innate and adaptive immune system that influences the outcome of evoked immune response. Briefly, the host immune cells are equipped with pathogen recognition receptors that include Toll‐like receptors (TLRs), complement receptors 1 and 2, C‐type lectin, B‐cell receptor (CD19) and T‐cell receptor (CD3) et cetera that recognise and form complementary binding regions with conserved molecules (antigen‐binding domains or epitopes and paratopes) often referred to as pathogen‐associated molecular patterns (PAMPs).^84^, ^85^ The PAMPs include surface or transmembrane molecules, intracellular antigens (proteins, mannoproteins, lipopolysaccharides, DNA and RNA).^84^

Briefly, during an immune activation, antigen presenting cells (dendritic cells, macrophages, monocytes or B cells) survey and pick up fungal antigens, phagocytose antigens, and present processed antigens to B cells and T cells.^38^, ^58^ In the process, cells of the innate immune system link their responses to cells of the adaptive immune system. Hence, the presented antigen cross links the T‐cell or B‐cell receptor to induce activation and stimulates polymerisation of the activation receptors (TLR, human leucocyte antigens (HLA‐DR), (CD21) et cetera) and the activation of co‐stimulatory receptors (CD80, CD86, CD28, et cetera) to form an activation synapse.^86^, ^87^, ^88^ Activation synapse guides activated signal to phosphorylate downstream of the activated cell to evoke an immune response. The produced immune response could be receptor activation (HLA‐DR, PD‐1, CD69 et cetera), cellular subset differentiation, antibody production (IgM (early infection), IgG and IgG class‐switched isotypes (IgG 1, 2, 3 or 4; later/reinfection), IgA (mucosal surfaces), cytokine response (Th‐1 cytokines; (IL‐1β, IFN‐γ, TNF‐α, Th‐2 cytokines; IL‐4, IL‐10, IL‐5, IL‐13, et cetera, Th‐17 cytokines; IL‐17, IL‐23 et cetera),^89^ chemokine receptor or chemokine ligands (CXCR3 in response to CXCL9 and CXCL10 stimulation, CXCR5 in response to CXCL13 stimulation et cetera)^90^, ^91^, ^92^, ^93^ or tolerance (anergy).

In this context, evasion of the CNS by *Cryptococcus* could overwhelm the system with aberrant immune activation and exuberant inflammation triggered by the fungal virulence antigens (melanin, polysaccharide capsule (GXM), production of enlarged titan fungal cells and other fungal virulence factors) induced by the fast replicating yeast during host evasion.^16^, ^38^, ^58^, ^66^, ^94^ The triggered cellular activation and pro‐inflammatory cytokine response (IFN‐γ, IL‐1β, IL‐6 and TNF‐ α et cetera) lead to an exuberant inflammation (cytokine storm) that manifests with raised intracranial pressure and meningoencephalitis.^25^, ^66^, ^95^

The *Cryptococcus* infection of the CNS induces inflow (trafficking) of intervening peripheral immune cells into the CNS, traversing the blood‐brain barrier. Alternatively, *Cryptococcus* infection of the CNS could induce local CNS cellular activation and differentiation to produce intrathecal activated and inflammatory response.^24^, ^25^, ^66^ The difference in immune response in cryptococcal meningitis between blood and the CSF compartments shows the existence of marked activation and increased inflammation in CSF than in blood at onset of cryptococcal meningitis and at onset of cryptococcosis‐IRIS.^24^, ^25^, ^66^ Moreover, aberrant immune activation and exuberant inflammation are associated with poor disease outcome in HIV‐associated *Cryptococcus neoformans* meningitis infection in Uganda.^32^, ^50^ Interestingly, the evoked cytokine response to *Cryptococcus* GXM can be manipulated in experimental setting.^96^, ^97^ But, to our knowledge, whether evoked immune response is different with infecting *Cryptococcus* species is not very clear. Moreover, unlike *C neoformans* sensu* lato* that causes disseminated infection, *C gattii* sensu* lato* establishes localised infection.^2^

The activation of PD‐1 expression may be a factor to modulate aberrant immune activation and exuberant inflammation in *Cryptococcus* infection. The observation that PD‐1 expression on plasmablasts/plasma cells is associated with HIV‐associated cryptococcosis mortality adds to the possible relevance of B cells in modulating the course of HIV‐associated cryptococcosis co‐infection to influence treatment outcome.^24^, ^98^ One study at cryptococcal meningitis diagnosis reported the PD‐1 expression on circulating cellular lineages at 2% of B cells (CD19 + lymphocytes), 25% of T cells and 1% of monocytes.^24^ Another study at the same timing reported PD‐1 expression on T cells at 60% of CD4 + T cells and 30% of CD8 + T cells with persistant high PD‐1 expression on circulating T cells beyond 12 weeks of follow‐up.^99^ In this case, it is not clear whether sustained PD‐1 expression in cryptococcosis is due to persistent activation or another mechanism. But, whether PD‐1 or its ligand expression is different with infecting *Cryptococcus* species is yet to the investigated.

In the HIV cohort, PD‐1 expression persisted on T cells beyond 22‐44 weeks of antiretroviral therapy.^65^ In compartments, PD‐1 is highly expressed in localised tissue infections that may be indicator altered immune response or pathogen immune escape mechanisms. Thus, PD‐1 though ubiquitous on immunocytes is predominantly expressed on T cells. The slow resolution of PD‐1 expression on effector cells may indicate either persistent activation, slow resolution of the evaded response or infecting *Cryptococcus* species specific.

## 7 BRAIN FIBROSIS IN CRYPTOCOCCAL MENINGITIS

Current literature is unclear on how fibrosis relates to cryptococcal meningitis and whether fibrosis influences cryptococcal infection and treatment outcomes are yet to be defined. Fibrosis resulting from collagen deposition on damaged vessels and tissues from the healing process following infection or injury leads to stiffening of tissues and impairment of function.^85^ The pro‐inflammatory cytokines (IFN‐γ, IL‐1β, IL‐6, TNF‐α, et cetera) and anti‐inflammatory cytokines (IL‐4, IL‐10 and TGF‐β) modulate the recovery process, but dysregulation of the these cytokine expression is demonstrated to influence the fibrosis.^85^, ^100^, ^101^, ^102^ Among cryptococcal meningitis patients, brain tissue fibrosis poses >50% risk of death.^102^

Postmortem studies among those who succumb to cryptococcal meningitis show that cryptococcomas are common in the brain and occlusions are prevalent in the subarachnoid space among 4%‐32% of those examined with arterial fibrosis and with infarction of the cerebellar (Figure 3).^102^ In addition, among cryptococcal meningitis patients, seizures occur in a third of the patients in sub‐Saharan Africa and Asia Pacific regions and that seizures increase the risk of death among these patients.^36^, ^103^ Interestingly, genetic studies examining the *C neoformans* sensu* stricto* clades show that differences in the clade has less influence on the intra‐*C neoformans* sensu* stricto* clades virulence.^3^, ^6^, ^7^, ^8^, ^10^, ^11^, ^12^, ^13^ But it is not clear whether virulence factors and induced immune responses are similar across *Cryptococcus* species complexes. This is important because of the limited diagnostics approaches in routine health care that can differentiate between *Cryptococcus* species that may influence treatment strategies amidst high residual 10%‐20% HIV‐associated cryptococcal meningitis‐related deaths with the optimal use of antifungals.

Other factor that can influence fibrosis in cryptococcal meningitis includes amphotericin B antifungal use induced phlebitis. Phlebitis though common and often regarded as local have undocumented systemic and brain adverse effects in cryptococcal meningitis treatment. How immune activation and inflammation resulting from HIV infection and/or cryptococcal meningitis relate to brain tissue fibrosis, hypoxia and infarction remains poorly understood. Inherently, during HIV infection, PD‐1/PD‐L1 upregulation predisposes the HIV‐infected host to arterial fibrosis.^65^ Moreover, induced PD‐1‐associated arterial stiffness persists for nearly a year.^65^ The PD‐1 pathway like the Th‐2 predominant immune response modulates the immune response resulting in shutting down of immune activation and inflammation leading to tolerance (immune non‐responsiveness) that may allow evading pathogen to thrive unchecked in the host.

Fibrosis may not be entirely bad, as may be used by the host as an attempt to limit the spread of infection. But fibrosis‐inducing factors may work in synergy with other *Cryptococcus* host susceptible factors like anaemia to impair oxygen supply to the vital organs like the heart and the brain (hypoxic hypoxia) and consequently impairing tissue survival. Hence, pathogens that survive in a hypoxic microenvironment like *Cryptococcus*, which evades and thrives in the macrophage, could take advantage of the induced hypoxia in the brain to survive while leading to fatal outcomes.^65^, ^102^, ^104^ Together, these host and pathogen factors that induce fibrosis could contribute to poor outcomes with cryptococcal meningitis.

**Figure 2:**
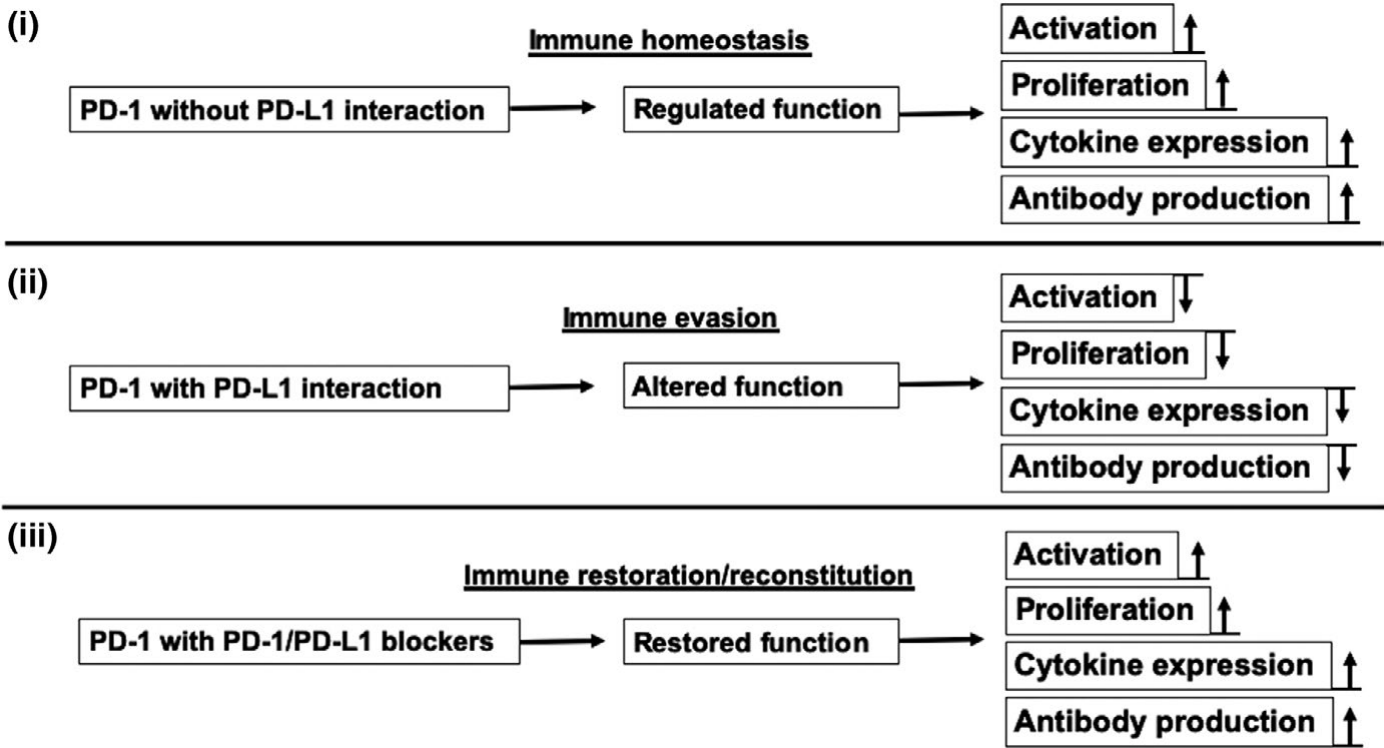
The PD‐1/PD‐L1 agonist/antagonist pathways. Up pointing arrows—upregulated cellular responses. Down pointing arrows—downregulated cellular responses. The mechanisms (i) occur in healthy, (ii) in an established infection and (iii) occur in the recovering infection or in the presence of anti‐PD‐1 antagonist interventions. PD‐1—programmed death‐1 ligand; PD‐L1—programmed death‐1 ligand

In other studies, the PD‐1/PD‐L1 axis‐induced tissue fibrosis is clearly demonstrated in the lungs ^101^ and brains of HIV‐infected patients.^105^ Together, brain tissue fibrosis (subarachnoid spaces and cerebellar) and arterial stiffness (anterior and posterior arteries)^102^ could limit the effectiveness of antifungal and antiretroviral drug penetration into the CNS. The limited drug entry to the CSF could, in turn, limit antigen clearance in those with arterial stiffness and may influence fungal recrudescence. Further induced brain tissue fibrosis could influence intracranial pressure. And exacerbated arterial stiffness could influence brain hypoxia and subsequent brain tissue infarction.^37^ It is plausible that targeted blood transfusion to increase blood oxygen carrying capacity especially to the brain, oxygen supplementation,^37^ use of anti‐PD‐1/PD‐L1 antagonists (PD‐1/PD‐L1 blockers) and adjunct therapy to decrease immune activation in combination with standard antifungal treatment could improve treatment outcomes in cryptococcal meningitis. Inherently, PD‐1 function is unaltered with immune status, (in health and in induced host immune suppression) as demonstrated in SIV macaque models of infection^24^, ^106^ that makes the PD‐1 pathway (Figure 2) amenable to a spectrum of disease conditions.

## 8 IMMUNE RECONSTITUTION INFLAMMATORY SYNDROME (IRIS)

The IRIS is a fatal condition associated with an exaggerated immune response to recall or persistent antigen following immune restoration with antiretroviral therapy. This is common among HIV‐infected patients with opportunistic co‐infections like cryptococcosis after they initiate ART.^25^, ^98^, ^107^ Thus, both *Cryptococcus* and or HIV infection may have profound effects on regulatory B‐cell responses in the setting where *C neoformans* sensu* lato* and interspecies hybrids cause most disease.^11^, ^12^, ^13^ And how regulatory B‐cell responses are reconstituted may influence immune activation and differentiation of both innate and adaptive immune response to impact on host recovery.^66^, ^108^

B cells are important regulators of the immune system.^80^, ^82^, ^100^, ^109^, ^110^, ^111^, ^112^, ^113^ Their roles include production of pro‐inflammatory cytokines, including IL‐6, TNF‐α and IFN‐γ,^114^ that may influence the extent of cryptococcosis‐induced inflammation, immune activation and host damage responses. Interestingly, studies that have attempted to harness regulatory B‐cell responses in chronic inflammatory disease conditions suggest that the quality of regulatory B cells rather than IL‐10 production is important in determining disease outcomes.^82^, ^113^, ^115^ This implies that the mechanism and the quality of the induced regulatory B‐cell function are more of cellular dependent and less reliant on the secreted soluble factors. Moreover, regulatory B‐cell responses are observed to be downregulated in HIV infection.^116^, ^117^, ^118^ And, factors that influence HIV infection and disease progression including bystander cell activation and HIV Nef‐induced bystander cell deaths may impair regulatory B‐cell function.^119^, ^120^ Thus, it is important that the role of regulatory B cell in cryptococcal meningitis and associated IRIS be defined as these cells may influence infection and disease outcome.

## 9 B‐CELL IMMUNE REGULATION

Regulatory B cells constitute about 10% of circulating B cells characterised by CD24 expression, IL‐10 production^80^ and production of transforming growth factor‐beta,^121^ in addition to IL‐35 and granzyme B production.^122^ Regulatory B cells share some marker homology with memory B cells (CD27^+^, CD38^+^, CD20^+^) and antibody‐producing B‐cell subsets (CD27^+^CD38^+^CD20) defined within the transitional B‐cell subsets (CD24^+^/CD38^+^), (CD24^+^/CD27^+^) and (IgM^+^/CD38^+^/CD1d^+^/CD147^+^) B cells.^112^, ^123^, ^124^ Other regulatory B cells include the IgG4^+^ anti‐allergen antibody expressing B‐cell phenotype; (CD25^+^/CD71^+^/CD73^+^).^82^ Thus, regulatory B cells constitute a well‐characterised cellular phenotype with a diverse pool of markers in humans and in animal models.

The factors required for ex vivo induction of IL‐10 production from regulatory B‐cell precursors include T‐cell co‐stimulation signals through: (a). CD28 or cytotoxic T lymphocyte‐associated protein‐4 for (CD80/CD86) interaction and co‐stimulatory receptor activation. (b). The CD40 co‐ligation. (c). The pro‐inflammatory cytokine milieu that modulates the microenvironment for regulatory B‐cell effector function modulated by the presence of type‐1 interferon family of cytokines; IFN‐α/β, IL‐1β, IL‐6, IL‐21 and or B cell‐activating factor‐F. (d). Inflammation induced by the presence of microbial factors either through complement receptor activating signals or through Toll‐like receptor‐9 signalling.^72^, ^83^ The presence of an antigen that cross links the B‐cell receptor induces downstream phosphorylation.^82^, ^125^ Thus, quantification of the regulatory B‐cell phenotypes in vitro is only a proxy for B‐cell immune regulatory response rather than an ex vivo host equated response. This is true as in vitro regulatory B cell‐induced IL‐10 production requires several exogenous manipulations that may not measure up to in vivo host response.^80^

Thus, a range of factors and mechanisms may influence HIV and associated cryptococcal meningitis co‐infection and treatment outcome. In health, potent vaccines and immune‐based therapies work by allowing target infection to establish below a threshold for disease onset as the effector response is evoked to control infection.^34^ This implies that immune regulatory mechanisms are integral to a balanced immune response to infectious disease with less fatal effects (minimal host damage) prior to resolution of infection (with/without treatment).^34^ We postulate that B‐cell immune modulatory mechanisms work in synergy with optimal treatment to regulate host damage responses to influence recovery.

The B‐cell subset developmental pathway may play a major role in the control of cryptococcal infection. B cells together with other immunocytes synergise T‐cell maturation. In an intact immune system, B cells are activated to produce antibodies. The B cells can activate naive T cells to aid in maturation of effector T cells. Moreover, during severe HIV immunosuppression, B cells and other immunocytes could become dysfunctional and unable to produce effector response that may influence immune reconstitution and host recovery.^20^, ^21^, ^22^, ^23^, ^30^, ^34^ Moreover, the defects in T‐cell activation and maturation are loosely linked to the onset of cryptococcal infection and associated disease outcomes.^118^, ^126^, ^127^

## 10 ANTIBODY RESPONSE IN CRYPTOCOCCAL MENINGITIS

In experiments to quantify *Cryptococcus*‐specific GXM antibodies among subjects with confirmed cryptococcal meningitis co‐infection by quantitative fungal culture, 45.7% of the subjects tested positive for *Cryptococcus*‐specific GXM IgG antibody.^128^ Interestingly, after acid treatment of the specimens to dissociate antibody from antigen‐bound immune complexes to release bound antibodies for measurement, 97.1% of subjects had detectable *Cryptococcus*‐specific GXM IgG antibody response.^128^ Thus, HIV and cryptococcosis co‐infected individuals demonstrate anti‐*Cryptococcus*‐specific antibody responses.^129^ However, the magnitude and the quality of antibody response may be inadequate especially at the onset of primary cryptococcosis to control the fast replicating fungus.^129^

In addition, the acute to early onset of cryptococcal meningitis from onset of symptoms could support the hypothesis that a protective antibody response in cryptococcosis occurs later (weeks to months following treatment) especially among index cases.^20^, ^45^ Additionally, the antibody response may be produced in later stages of infection when the host may be already overwhelmed with the infection. This may render antibody response less beneficial to the debilitated host as most of the produced antibody response may end up being bound to immune complexes and to the polysaccharide capsule.^128^ Conversely, it could be interpreted that index cryptococcosis infection (without circulating protective antibodies) causes fatal disease in the susceptible host and not latent cryptococcosis (with circulating specific antibodies). This could be true since both the latent and new *Cryptococcus* infection cause cryptococcal meningitis.^5^, ^60^

## 11 REGULATION OF B‐CELL TRAFFICKING IN INFLAMMATION

The immune cells extravasate the peripheral and lymphatic circulation to survey for antigens in the tissues and extra cellular spaces. This movement of immune cells from circulation to the tissues is important and enables responsive immune cells to effect their activated responses.^130^ Following cellular activation, responsive immune cells induce chemokine receptor expression and production of adhesion molecules. The induced chemokine receptor and adhesion molecules bind ligand to facilitate cellular chemoattraction through an inducing signal gradient in the target site.^131^, ^132^

The chemokine receptor CXCR5 and its ligand CXCL13 recruit naïve activated B cells to the follicles of the germinal centres, resulting in B‐cell differentiation to plasma cells.^133^ The induction of CXCR5 and CCR7 receptors on B cells further activates responsive naïve B cells to migrate and localise in secondary and tertiary lymphoid tissues including the lymph node, Peyer's patches, mucosal and gut‐associates lymphoid tissues for further maturation.^134^, ^135^ Seeding of activated immune cells to the inflammatory sites leads to formation of ectopic (tertiary) germinal centres that surround inflamed tissues.^136^ Other B‐cell homing receptors to the inflammatory sites include expression of CXCR3 receptors in response to CCL9, CCL10 ligand induction. The response may be in addition to inducing interferon‐inducible protein‐10 (IP‐10) and CCL11, which are associated with B‐cell trafficking to the CNS in neuropathies.^137^, ^138^

Meya *et al*, have shown that immune cell homing and localisation in the CSF are possible during HIV and associated cryptococcosis co‐infection.^66^ In this study, CD4 T‐cell frequency was low at onset of HIV‐associated cryptococcosis among individuals who later developed cryptococcal IRIS. The onset of IRIS was associated with the later increase in the CD4 T‐cell frequency in the CSF.^66^ A study in a similar setting showed a wide spectrum of pro‐inflammatory protein profile (IFN‐γ, IL‐1β, TNF‐α) and anti‐inflammatory proteins (IL‐10, granulocyte monocytes/macrophage colony stimulating factor (GM‐CSF)) being upregulated in CSF during cryptococcal infection.^25^ This array of immune modulators in CSF may show the importance of cellular trafficking or passive entry of vital molecules to the CSF in response to intrathecal stimulations. Some of the markers expressed could exacerbate inflammation and immune activation of host damage responses that could have a negative influence on cryptococcal disease and treatment outcomes.^21^ Studies in cancer therapy, organ transplant recipients and chronic inflammatory disease conditions show that regulation of these chemokine receptor expression (CXCR3 and CXCR5) by blocking their ligand interaction modulated host recovery.^139^, ^140^, ^141^ Thus, evaluation of the role and relevance of these B‐cell chemokine receptors expression induction (CXCR3, CXCR5, CCR7 and CCR3) in CSF and blood may advance our understanding of HIV and cryptococcosis pathogenesis and potentially treatment outcome paradigms for translation.

## 12 FACTORS LINKED TO FATAL *CRYPTOCOCCUS* CENTRAL NERVOUS SYSTEM INFECTION

**Figure 3:**
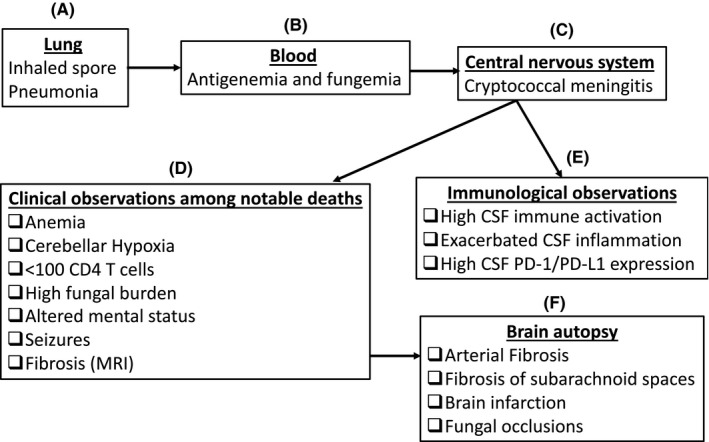
Model of fatal cryptococcal infection irreversible with current optimal antifungal therapy among some HIV and cryptococcal meningitis co‐infected patients. A, The subclinical lung infection may resolve without symptoms or may persist with latent infection. B, Blood infection indicated systemic spread. C, Central nervous system infection indicates onset of often fatal disease, cryptococcal meningitis. D, Clinical observations linked to poor survival outcome. E, Clinical observations associated with mostly CSF infection. F, Factors observed at autopsy

Among patients with HIV‐associated cryptococcal meningitis where *C neoformans* sensu* lato* and interspecies hybrids cause most disease,^11^, ^12^, ^13^ inconsistent variables (Figure 3D) that influence disease outcome have been reported including early ART initiation among HIV and ART naïve subjects.^20^ The high fungal burden and altered mental status at cryptococcal meningitis diagnosis coupled with the slow fungal clearance following antifungal therapy.^21^, ^50^ The prevalence of seizures at diagnosis and the incidence seizures during the course of treatment and follow‐up among individuals with above 96 000 fungal colony‐forming units that is 3‐four fold higher in those with seizures compared to those without seizures.^36^ Limited oxygen blood carrying capacity (anaemia) potentially leading to low brain oxygen saturation, hypoxia.^37^, ^142^ Although amphotericin B is known to suppress erythropoietin,a factor that may influence the extent of anaemia in cryptococcosis sequelae.^143^ Surprisingly, amphotericin B‐induced anaemia is not associated with cryptococcosis cause fatality in first two and half months of cryptococcosis diagnosis.^142^ And fibrosis among cases of cryptococcal meningitis may influence onset and the extent of intracranial pressure build up, hypoxia, brain infarction and antifungal drug penetration.^102^ Interestingly, these variables and factors associated with poor cryptococcosis outcome are reported independent of each other within similar large cohorts of HIV‐associated cryptococcal meningitis.^20^, ^21^, ^36^, ^37^, ^50^, ^102^ This implies that multiple or slightly independent pathways drive host pathology. Thus, advances to alter poor survival outcomes could require deliberate effects to combine multiple factors in combined interventions targeting multiple pathways in the host.

In addition, an ineffectual immune system with a skewed Th‐2 immune response has been associated with poor disease outcomes. The Th‐2 immune response is seen to inhibit activation of *Cryptococcus*‐specific antifungal pro‐inflammatory cytokine responses, thus impairing fungal clearance.^50^ And the high frequency of pro‐inflammatory cytokines responses (IFN‐γ, TNF‐α and IL‐12) coupled with a high frequency of CSF infiltrating white blood cells after ART initiation is associated with a risk of cryptococcal meningitis‐immune reconstitution inflammatory syndrome (CM‐IRIS).^107^

But, predominance of a pro‐inflammatory cytokine response alone is postulated to work in synergy with antifungal treatment to enhance fungal clearance.^20^, ^21^ Conversely, in animal models of cryptococcal infection, animals that produce antibodies specific to *Cryptococcus* virulence factors including melanin, urease and polysaccharide capsule after exposure to pulmonary cryptococcal antigens demonstrate higher numbers of long‐term survivors. This was observed following a challenge with a live lethal dose of *Cryptococcus* in experimental animals.^61^, ^64^, ^144^, ^145^, ^146^ Similarly, high expression of PD‐1 on plasmablasts/plasma cells is linked to host survival among patients with cryptococcosis.^24^

Thus, survival in HIV and cryptococcosis co‐infection is associated with higher expression of pro‐inflammatory cytokine responses (IFN‐γ, TNF‐α and IL‐6) and corresponding higher influx of white blood cells that infiltrate the cerebrospinal fluid.^20^, ^21^, ^49^ Although unregulated expression of these cytokines is linked with onset of IRIS, their production seems to function in synergy with antifungal treatment to enhance fungal clearance^20^, ^21^, ^49^ while downregulation of pro‐inflammatory cytokine responses is associated with poor control of fungal antigens and mortality.^50^ At cryptococcosis diagnosis, individuals at risk of IRIS sequalae have higher expression of IL‐6 and IL‐10.^147^ In addition, these individuals have higher expression of anti‐glucuronoxylomannan antibodies^148^ and overexpression of pro‐inflammatory cytokines.^97^, ^149^ Moreover, IL‐6 expression, one of the upregulated pro‐inflammatory cytokine, is known to influence the capability of B cells to proliferate.^150^ Thus, possibly linking B cells to the onset of IRIS.

## 13 CONCLUSION AND PERSPECTIVES

Despite four decades of HIV era and advances to treat those infected with optimal therapy, cryptococcal meningitis remains a significant contributor of death among HIV‐infected adults especially in sub‐Saharan Africa. Consolidated new insights show that the HIV and cryptococcal meningitis‐associated poor survival outcome factors are associated with fungal induced neuropathies related to the inducers of seizures, altered mental status, brain tissue fibrosis and brain hypoxia and potentially the involved cryptococcal species. In addition, altered immune response involving PD‐1/PD‐L1 pathway may dysregulate immune activation to influence inflammation and impact on brain fibrosis and brain infarction. And dysregulated erythropoiesis associated with prevalent anaemia together with induced fibrosis may influence hypoxia and brain infarction. These insights come at a time of unknown immunological target to prevent HIV‐associated cryptococcal meningitis and adequate therapy to prevent mortality. Together, the eluded factors and underlying mechanisms are hypotheses generating and will guide further studies to finding targets to prevent infection or adequate therapy to prevent mortality. Interventions like the use of supplemental oxygen, blood transfusion to boost blood oxygen carrying capacity and the use of anti‐PD‐1/PD‐L1 blockers to restore appropriate immune reconstitution may be valuable advances to alter poor treatment outcome.

## 14 LIMITATIONS

Our findings are without important limitations that our discussion is based on online published literature generalised to *Cryptococcus* in the setting of HIV‐associated cryptococcosis. However, individual *Cryptococcus* species may influence infection or disease recovery differently requiring species identification to define species‐specific pathologies and treatment outcome for clinical relevance. This is important because *Cryptococcus* still causes substantial morbidity and mortality that may require different treatment strategies to prevent residual 10%‐20% of cryptococcosis‐related deaths occurring with optimal use of antifungals. And that, although we conducted a wide literature search, we might have left out relevant literature. And some deductions are based on a few articles on the subject matter that may limit generalisation.

## CONFLICT OF INTEREST

Nothing to disclose.

## AUTHOR CONTRIBUTION

**Samuel Okurut:** Conceptualization (lead); Data curation (lead); Formal analysis (lead); Funding acquisition (lead); Investigation (lead); Methodology (lead); Project administration (lead); Resources (lead); Software (lead); Supervision (lead); Validation (lead); Visualization (lead); Writing‐original draft (lead); Writing‐review & editing (lead). **Joseph Olobo:** Conceptualization (supporting); Data curation (supporting); Formal analysis (supporting); Investigation (supporting); Methodology (supporting); Project administration (supporting); Supervision (lead); Validation (lead); Visualization (lead); Writing‐original draft (lead); Writing‐review & editing (lead). **David Boulware:** Conceptualization (equal); Data curation (lead); Formal analysis (supporting); Funding acquisition (supporting); Investigation (lead); Methodology (supporting); Project administration (lead); Resources (supporting); Software (supporting); Supervision (supporting); Validation (supporting); Visualization (lead); Writing‐original draft (lead); Writing‐review & editing (lead). **David Meya:** Conceptualization (equal); Data curation (equal); Formal analysis (supporting); Funding acquisition (supporting); Investigation (lead); Methodology (equal); Project administration (lead); Resources (supporting); Software (supporting); Supervision (lead); Validation (lead); Visualization (lead); Writing‐original draft (lead); Writing‐review & editing (lead).

## ETHICAL CONSIDERATIONS

Nothing to disclose.
